# Effects of HIV infection on maternal and neonatal health in southern Mozambique: A prospective cohort study after a decade of antiretroviral drugs roll out

**DOI:** 10.1371/journal.pone.0178134

**Published:** 2017-06-02

**Authors:** Raquel González, María Rupérez, Esperança Sevene, Anifa Vala, Sónia Maculuve, Helder Bulo, Arsénio Nhacolo, Alfredo Mayor, John J. Aponte, Eusébio Macete, Clara Menendez

**Affiliations:** 1 ISGlobal, Barcelona Ctr. Int. Health Res. (CRESIB), Hospital Clínic-Universitat de Barcelona, Barcelona, Spain; 2 Manhiça Health Research Center (CISM), Manhiça, Mozambique; 3 Eduardo Mondlane University, Faculty of medicine, Maputo, Mozambique; Universidade de Sao Paulo Instituto de Ciencias Biomedicas, BRAZIL

## Abstract

**Introduction:**

The HIV epidemic is concentrated in sub-Saharan Africa. However, limited information exists on its impact on women and infant’s health since the introduction of antiretroviral drugs in this region, where health resources are often scarce.

**Methods:**

The effect of HIV infection on maternal health, birth outcomes and infant health was analysed in two contemporary cohorts of HIV-uninfected and HIV-infected pregnant women from southern Mozambique. Pregnant women attending the first antenatal care visit were followed until one month after delivery. Antiretroviral therapy was administered based on CD4+T cell count and clinical stage. Maternal and neonatal morbidity and mortality, as well as pregnancy outcomes were assessed by mother’s HIV status.

**Results:**

A total of 1183 HIV-uninfected and 561 HIV-infected pregnant women were enrolled. HIV-infected women were more likely to have anaemia both at the first antenatal care visit and at delivery than HIV-uninfected women (71.5% versus 54.8% and 49.4% versus 40.6%, respectively, p<0.001). Incidence of hospital admissions during pregnancy was increased among HIV-infected women (RR, 2.04, [95%CI, 1.45; 2.86]; p<0.001). At delivery, 21% of HIV-infected women reported being on antiretroviral therapy, and 70% having received antiretroviral drugs for prevention of mother to child transmission of HIV. The risk of stillbirths was doubled in HIV-infected women (RR, 2.16 [95%CI 1.17; 3.96], p = 0.013). Foetal anaemia was also increased among infants born to HIV-infected women (10.6% versus 7.3%, p = 0.022). No differences were found in mean birth weight, malaria, prematurity and maternal and neonatal deaths between groups.

**Conclusions:**

HIV infection continues to be associated with significant maternal morbidity and poor neonatal health outcomes. Efforts should urgently be made to identify the barriers that impede improvements on the devastating effects of HIV in African women and their infants.

**Trial registration:**

ClinicalTrials.gov NCT 00811421.

## Introduction

Approximately 37 million people were estimated to be living with HIV in the world at the end of 2015[1]. The centre of the global pandemic is concentrated in sub-Saharan Africa (SSA), region that accounts for two thirds of the global total of new HIV infections. Women of reproductive age are disproportionately impacted by the infection which constitutes a significant cause of maternal morbidity and mortality [2]. Notably, it is estimated that every hour 50 women are infected with HIV in the world [3]. Besides, although antiretroviral (ARV) drugs have been rolled out free of charge through the public health system in recent years, their access is often limited and delayed leading to significant rates of mother-to child transmission (MTCT) of HIV[4]. Globally, the annual number of newly infected children in 2014 was 220 000 and the highest burden of them concentrated in SSA[1].

Maternal HIV infection in women who have not received antiretroviral therapy has been associated with adverse pregnancy outcomes such as preterm birth, low birth weight (LBW), small for gestational age (SGA) and stillbirth, especially in SSA[5]. Importantly, preterm birth constitutes the second world’s leading cause of death in children under five years[6]. In 2010, it was estimated that 32·4 million infants were born SGA in low-income and middle-income countries (27% of live births), of whom 10·6 million infants were born at term and LBW [7].

The effects of HIV infection in pregnancy have been mainly studied in high income countries, where both disease burden and health systems are significantly different compared with those in the African region [8]. Several studies conducted in SSA have reported that HIV-infected women are at increased risk of maternal anaemia and adverse pregnancy outcomes such as stillbirth, LBW and preterm new-borns [9–11]. In addition, children born to HIV-infected mothers are at increased risk of mortality regardless of their HIV infectious status [12–14].

Establishing the impact of HIV infection on maternal and infant health is particularly challenging in SSA because of the presence of factors associated with both HIV infection and adverse pregnancy outcomes such as malnutrition, anaemia and other frequent concurrent infections such as syphilis and malaria [15]. It has been estimated that approximately one million pregnancies per year are co-infected with malaria and HIV in SSA [16]. Because of the presence of the aforementioned confounding factors it is likely that the impact of HIV infection on pregnancy outcomes is more significant in women from low income countries compared to those from developed regions [15]. This work attempts to contribute to the knowledge of the effects of maternal HIV infection on pregnancy outcomes and infant’s health in SSA countries after a decade of ARV drugs roll-out.

Mozambique is one of the sub-Saharan countries that concentrates the highest burden of HIV infection with approximately 1.5 million people living with HIV in 2015[17]. Official estimates from the first national survey estimated a HIV prevalence in 2009 of 15% (95%CI 13.9;16) in individuals aged 15–49 years old, reaching over 19% in the southern region of the country[18]. As in neighbouring countries, Mozambican women of reproductive age carry the brunt of the disease, with an HIV prevalence among pregnant women of nearly 30% in some areas of the country [19, 20]. In 2013 the country adopted the latest WHO guidelines to prevent MTCT of HIV of providing life-long ARVs to pregnant women regardless of their immunological status (option B+)[21]. Given the high HIV burden in women of reproductive age it becomes critical to determine its impact on both maternal and infant’s health to help guiding intervention strategies. Importantly, limited data exist on epidemiological features of maternal HIV infection since 2004, when free large scale-up of ARV drugs was initiated in the country [22]. The aim of this study was thus to describe the effects of HIV infection on maternal health, birth outcomes and neonatal survival in southern Mozambique after a decade of ARVs roll-out.

## Materials and methods

### Study population and setting

The study was conducted at the *Centro de Investigação em Saúde de Manhiça* (CISM) and the Manhiça District Hospital (MDH), located in a semi-rural area from southern Mozambique. Since 1996 the CISM conducts continuous demographic surveillance in the Manhiça district covering a population of 92000 inhabitants [23]. The voluntary counselling and testing (VCT) programme for prevention of MTCT of HIV was integrated into routine practice at the MDH antenatal clinic (ANC) in July 2003. The prevalence of HIV infection in pregnant women attending the ANC in 2010 was 29% [19]. Eligible study participants were pregnant women of all gravidities attending the study ANC for the first time with a gestational age ≤ 28 weeks (refer to S1 Text for further information). At the time of the study, prevention of MTCT (PMTCT) of HIV relied on antenatal administration of daily monotherapy with zidovudine (AZT) to the mother from 14 weeks of gestation, combined ARVs during labour and up to one week postpartum (single dose Nevirapine [sd-NVP] and daily AZT plus lamivudine [3TC]), together with the administration of daily NVP to the infant, from birth until one week after weaning. Antiretroviral therapy (ART) was recommended when CD4+T cell count decreased to levels <350 and/or when the woman was in 3 or 4 HIV/AIDS WHO clinical stage and ARVs were delivered to pregnant women at the monthly ANC clinic visits[24].

### Study design

This study is part of two concurrent randomized controlled trials (NCT00811421) that evaluated mefloquine for malaria prevention in HIV-uninfected and HIV-infected pregnant women. The two trials ran in parallel at the same ANC and maternity wards and were conducted by the same study personnel. Details of the trials design and procedures are given elsewhere [25, 26]. In brief, after giving informed consent, eligible pregnant women with a gestational age ≤ 28 weeks (assessed by bimanual palpation of fundal height measurement using a metric tape) and living in the CISM study area, were invited to participate (see S1 Text). The only different inclusion criterion to be enrolled into one or other trial was the HIV status. Enrolment of pregnant women was conducted from March 2010 to April 2012 and follow-up was completed in February 2013.

### Ethical considerations

Study protocols and informed consent forms of the trials were reviewed and approved by the Ethics Committees from the Hospital Clínic of Barcelona (Spain) and the National Ethics Review Committee from Mozambique. The trials were conducted under the provisions of the Declaration of Helsinki and in accordance with Good Clinical Practices guidelines set up by the WHO and by the International Conference on Harmonization.

### Follow up and sample collection

At first ANC visit, HIV serostatus, haemoglobin and the syphilis rapid plasma reagin test (RPR) were assessed on fingerprick collected capillary blood. All women received a long lasting insecticide treated net and antimalarials for prevention of malaria as part of trial’s interventions (either IPTp-SP or IPTp-mefloquine those HIV-uninfected; either cotrimoxazole or cotrimoxazole plus IPTp-mefloquine those HIV-infected) [25, 26]. In HIV-infected pregnant women venous blood was taken for CD4+T cell count and viral load determination. Women were visited monthly at the ANC clinic and were encouraged to attend the study health facility whenever they had any health complaint. A health facility-based passive surveillance system was established to capture unscheduled visits of participants during follow-up. At delivery, a sample from the mother’s peripheral blood was collected for haemoglobin, CD4+T cell count, HIV viral load and malaria infection evaluation; cord blood and placental samples were also taken. Twins were not excluded from the study. One month after the end of pregnancy, a capillary blood sample from the mother was collected for malaria parasite determination. Infants were weighed and measured at birth (including head circumference measurement) and followed until four weeks of age to assess survival and general morbidity. At this visit, they were weighed and measured, their nutritional status was assessed and a capillary blood sample was collected for HIV DNA PCR analysis to determine the proportion of MTCT of HIV.

### Laboratory methods

HIV serostatus was assessed using rapid test (Determine, Abbot Laboratories, USA), and positive results confirmed using Unigold rapid test (TM HIV, Trinity Biotech, Ireland), following national guidelines at the first ANC visit. CD4+T cell count was determined by flow cytometry. HIV viral load was determined from plasma cryopreserved at -80°C using the COBAS AMPLICOR or AmpliPrep (Roche Diagnostics, Rotkreuz, Switzerland) devices; these assays have a lower detection limit ranging from 50 to 400 copies/mL. Haemoglobin levels were determined using mobile devices (HemoCue [www.eurotrol.com]) on capillary blood samples. *Plasmodium falciparum (P*. *falciparum)* parasites were identified by microscopy on Giemsa-stained blood films according to standard, quality-controlled procedures [27, 28], [29]. Placental biopsies and impression smears were processed, stained and examined following standard procedures [30, 31].

### Data management, statistical methods and definitions

Data were double-entered using the OpenClinica Enterprise software for clinical data management (www.openclinica.com). Peripheral malaria infection was defined as the presence of asexual *P*. *falciparum* parasites of any density in a blood smear. Placental infection was defined as the presence of parasites and/or pigment detected by histological examination or impression smears [30]. Maternal anaemia was defined as an haemoglobin level < 11g/dL and severe anaemia as haemoglobin< 7 g/dL, while foetal anaemia was defined as a cord blood haemoglobin level <12.5 g/dl. A clinical malaria episode was defined as presence of *P*. *falciparum* parasites in a blood smear plus any sign or symptom suggestive of malaria including: current fever (axillary temperature ≥ 37.5°C) or history of fever in the last 24 hours, and/or pallor, and/or arthromyalgias and/or headache, and/or history of convulsions [32]. Low birth weight (LBW) was defined by a weigh of less than 2500 grams at birth. Stillbirth was defined as a foetal death occurring after 20 complete weeks of gestation, and miscarriage as the termination of pregnancy and expulsion of an embryo or a foetus prior to 20 complete weeks of gestation. Neonatal death refers to the death of a live-born baby within the first 28 days of life. Quantitative variables were treated either as continuous or categorical. For instance, CD4+T cell count was categorized as ≤ 350 or > 350 cells/μl. Maternal viral load was analysed in the logarithmic scale using censored regression (Tobit regression) including as censored values those that were lower than 400 copies/mL[33, 34]. Z score was calculated for anthropometric index to assess nutrition in infants (weight for height), according to WHO standard definitions[35].

Proportions for categorical variables were assessed using the chi-square test or Fisher’s exact test where appropriate. The Student’s t test was used to compare means and medians of continuous variables according to variable characteristics. Only records with information on outcome of interest were analysed. Incidences of all-cause hospital admissions and all-cause outpatient attendance during pregnancy were analysed using negative binomial regression. The incidence of clinical malaria episodes was compared between HIV-infected and uninfected women using a negative binomial regression allowing for interdependence between episodes within the same subject, excluding from the time at risk the 28 days after the end of treatment of a malaria episode. The proportion of women with adverse pregnancy outcomes was compared by HIV-status using a modified binomial regression [36]. These analyses were done unadjusted and adjusted by baseline significant variables (age, gestational age, gravidity, RPR, anaemia and literacy, study intervention) and clinically relevant factors depending on the outcome for control of confounding factors. Incidences of hospital admissions in the neonate were also analysed using negative binomial regression. All statistical tests were two-tailed and statistical significance was defined as p<0.05. Data analysis was performed using Stata version 13 (Stata Corp., College Station, TX, US).

## Results

**Fig 1:**
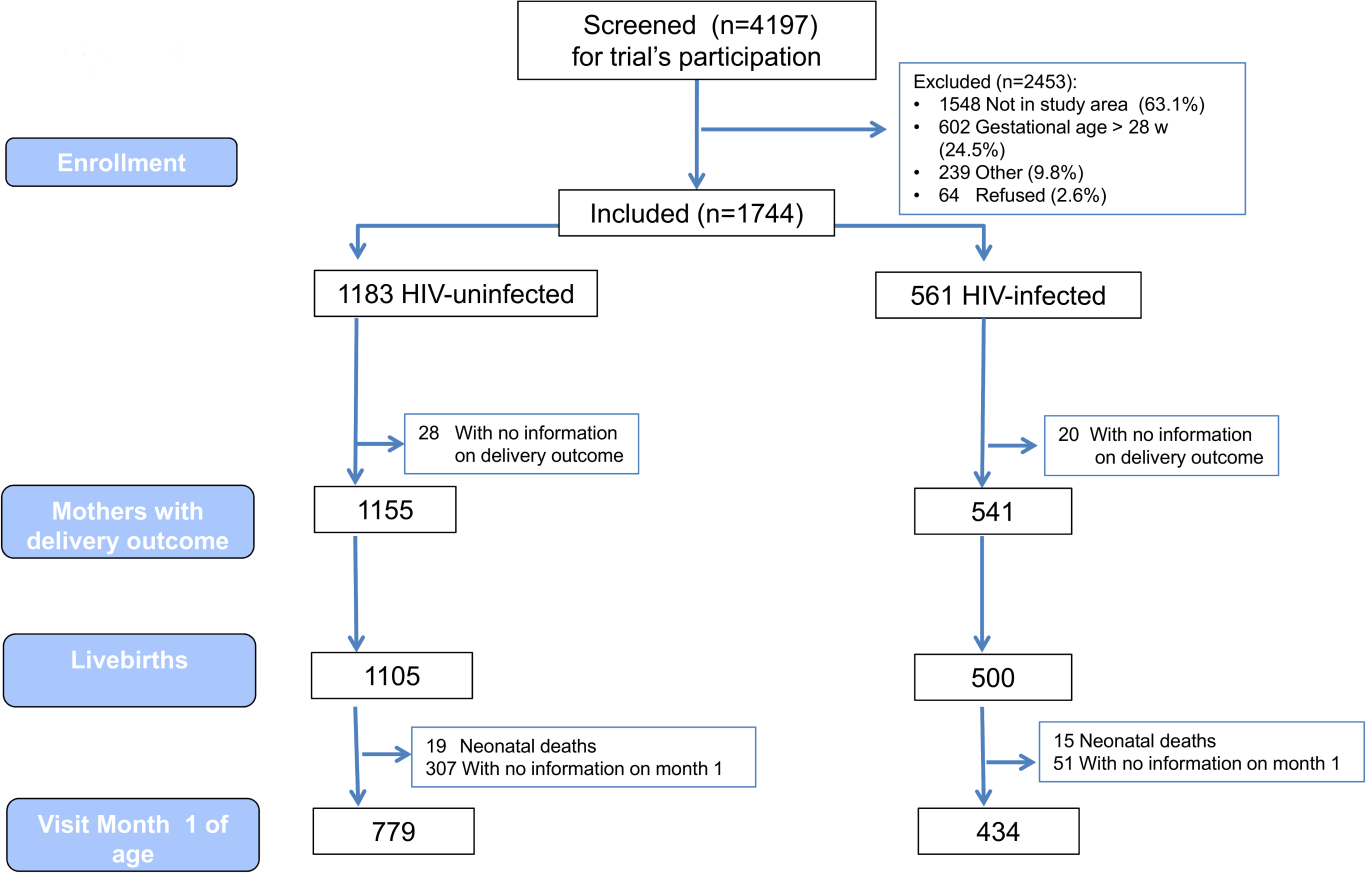
Study flow.

From the 4197 pregnant women screened for participation in the two trials, 64 (1.5%) refused to be enrolled in the studies (Fig 1). A total of 1744 pregnant women participated in the two trials and contributed to this analysis; 32% of participants (n = 561) were HIV-infected. No consent withdrawals occurred during follow-up. A total of 1155 HIV-uninfected women (out of 1183) and 541 HIV-infected (out of 561) had information on pregnancy outcome. These figures represent an overall loss to follow up frequency of 2.8% during pregnancy.

### Characteristics at first antenatal care visit

Comparison of baseline characteristics of pregnant women by HIV status is summarized in Table 1. On average, HIV-infected women were older than those HIV-uninfected (mean age of 27.0 and 23.5 years respectively, p<0.001). Mean gestational age was lower (20.2 weeks) in HIV-infected than HIV-uninfected (21.0 weeks) women (p<0.001). In addition, the proportion of multigravida women (88.2% *versus* 63.9%), the prevalence of maternal anaemia (71.5% *versus* 54.8%) and severe anaemia (4.1% *versus* 1.8%) were significantly higher in HIV-infected than in HIV-uninfected women (p<0.001, Table 1). No differences were found in the proportion of malnourished women between the two groups (Table 1). The prevalence of syphilis was higher in HIV-infected women (6.6%) than in HIV-uninfected women (1.8%, p<0.001).

**Table 1:** Characteristics of study participants at baseline.

Variables		HIV-uninfected	HIV-infected	p- value*
Participants ^1^		1183 (67.8)	561 (32.2)	
Age (years) ^2^		23.5 (6.7) [1182]	27.0 (6.0) [561]	**<0.001**
Gravidity (categories) ^1^	Primigravidae	427 (36.1)	65 (11.6)	
	1–3 previous pregnancies	560 (47.3)	367 (65.4)	
	4 or more pregnancies	196 (16.6)	128 (22.8)	**<0.001**
	No data	0 (0)	1 (0.2)	
Weight (kg) ^2^		61.1 (9.2) [1183]	60.5 (8.1) [561]	0.214
Height (cm) ^2^		158.9(7.4) [1181]	159.4 (6.6) [561]	0.090
MUAC (cm) ^2^^,^^3^		26.8 (3.0) [1177]	26.7 (2.6) [555]	0.599
Malnutrition ^4^		35 (3.0)	11 (2.0)	0.225
Gestational Age (weeks) ^2^		21.0 (5.1) [1182]	20.2 (5.4) [561]	**0.004**
Gestational Age in categories ^1^	First Trimester	68 (5.8)	49 (8.7)	
	Second Trimester	778 (65.8)	374 (66.7)	
	Third Trimester	336 (28.4)	138 (24.6)	0.055
	No data	1 (0.1)	0 (0)	
Literate (can read and/or write) ^1^	No	219 (18.5)	169 (30.1)	**<0.001**
	Yes	964 (81.5)	392 (69.9)	
Syphilis test ^1^	Positive	21 (1.8)	37 (6.6)	**<0.001**
	Negative	1158 (97.9)	521 (93.4)	
	No data	4 (0.3)	0 (0)	
Haemoglobin (g/dL) ^2^		10.7 (1.7) [1175]	10.1 (1.6) [559]	**<0.001**
Anaemia (Hb < 11g/dL) ^1^		648 (54.8)	401 (71.5)	**<0.001**
Severe anaemia (Hb < 7g/dL)		21 (1.8)	23 (4.1)	**<0.001**

* Proportions were compared using the chi-square test and continuous variables with the Student’s t test

^1^n (column percentage)

^2^Arithmetic Mean (SD)[n]

^3^MUAC: Middle Upper Arm Circumference

^4^ Malnutrition is defined as a MUAC≤22 cm

Twenty four per cent of HIV-infected pregnant women reported ARV drug use at first ANC visit, either as therapy or PMTCT in the current pregnancy (Table 2).

**Table 2:** Viral load and CD4 cell counts of HIV-infected women.

Parameter		Baseline	Delivery	P-value*
Mean Viral load (copies/mL)^1^		78708.9 (253445) [509]	52895.3 (270570) [484]	<0.001
Viral Load categories (copies/mL) ^2^	Undetectable	79 (14.1)	142 (25.3)	<0.001
	400–999	142 (25.3)	29 (5.2)	
	1000–9999	186 (33.2)	266 (47.4)	
	>9999	102 (18.2)	47 (8.4)	
	No data	52 (9.3)	77 (13.7)	
Mean CD4+ T cells count (c/μL)		466.4 (267.4) [525]	431.3 (307.5) [432]	0.013
CD4+ T cells count categories (c/μL) ^2^	≤350	188 (33.5)	205 (36.5)	<0.001
	>350	337 (60.1)	227 (40.5)	
	No data	36 (6.4)	129 (23)	
On ARV^3^		130 (24.0)	464 (91.0)	<0.001

* By Student’s t test for paired comparisons or Chi-square test

^1^Arithmetic Mean (SD)[n]

^2^n (column percentage)

^3^ Self-reported use of ARV: administered either as therapy or PMTCT.

### Maternal morbidity and pregnancy outcomes

No differences were found between groups in the incidences of clinical malaria and all-cause outpatient attendance during pregnancy (Table 3). In contrast, the incidence of all-cause and non-obstetric hospital admissions was twice as high in HIV-infected compared to HIV-uninfected women (Relative Rate, RR, 2.04 [95%CI, 1.45; 2.86]; p<0.001) and (RR, 1.94 [95%CI, 1.38; 2.73]; p<0.001), respectively (Table 3).

**Table 3:** Maternal and neonatal morbidity by mothers HIV status.

Incidences	HIV-uninfected	HIV- infected	Relative Rate	95%CI	p- value
Outpatient visits during pregnancy					
N/PYAR	313/616.68	159/284.81	1.07^a^	0.89–1.30	0.480
Incidence	0.51	0.56	1.03^b^	0.84–1.27	0.353
Clinical malaria in pregnancy					
N/PYAR	26/521.72	10/239.33	0.81^a^	0.39–1.68	0.574
Incidence	0.05	0.042	0.65^b^	0.30–1.44	0.447
Hospital admissions					
N/PYAR	86/528.55	81/246.88	1.98^a^	1.466–2.69	**<0.001**
Incidence	0.16	0.33	2.04^b^	1.45–2.86	**<0.001**
Non-obstetric hospital admissions					
N/PYAR	81/528.19	78/246.88	1.91^a^	1.41–2.60	**<0.001**
Incidence	0.15	0.32	1.94^b^	1.38–2.73	**<0.001**
Neonatal hospital admissions					
N/PYAR	62/83.94	22/45.92	0.761^a^	0.468–1.237	0.271
Incidence	0.74	0.48	0.765^c^	0.470–1.244	0.280

^a^Unadjusted analysis

^b^Adjusted analysis by baseline variables (age, literacy, gravidity, RPR and anaemia) and study intervention

^c^ Adjusted analysis by low birth weight; N = number of episodes; PYAR: person/year at risk; p-value from negative binomial regression model using Wald test. A clinical malaria episode was defined as presence of *P*. *falciparum* parasites in a blood smear plus any sign or symptom suggestive of malaria including: current fever (axillary temperature ≥ 37.5°C) or history of fever in the last 24 hours, and/or pallor, and/or arthromyalgias and/or headache, and/or history of convulsions.

The proportion of women with anaemia and severe anaemia at delivery was also higher in HIV- infected (49.4% and 4.1%, p<0.001, respectively) than in those HIV-uninfected (40.6% and 1.8%, p = 0.004, respectively; Table 4). On the other hand, no differences were found in the prevalence of peripheral *P*. *falciparum* parasitemia at delivery and placental malaria between the two groups (Table 4). The proportion of miscarriages was higher in HIV-infected women (2.2%) than in those HIV-uninfected (1.7%), but this difference was not statistically significant (p = 0.492). There were significantly more stillbirths in HIV-infected women (5%) than in HIV-uninfected women (2.6%, p = 0.011). The RR of stillbirths in HIV-infected women compared with HIV-uninfected was 1.97 ([95%CI 1.17–3.29], p = 0.010) and 3.34 ([95%CI 1.80–6.20], p<0.001 in the unadjusted and adjusted analysis, respectively (Table 4). No differences were found in the proportion of maternal deaths between the two groups (Table 4).

**Table 4:** Maternal outcomes by HIV status.

Parameter		HIV-uninfected (N = 1155)	HIV-infected (N = 541)	p- value*	Relative Rate	95%CI
Delivery setting^1^						
	Study health facility	965 (83.6)	440 (81.3)			
	Other health facility	57 (4.9)	24 (4.4)	0.157		
	Home	77 (6.7)	36 (6.6)			
	Other	56 (4.9)	41 (7.6)			
Mode of delivery ^1^						
	Vaginal	1048 (92.9)	497 (94.0)	0.430		
	Cesarean	80 (7.1)	32 (6.0)			
Anaemia at delivery (<11 g/dL) ^1^		446 (40.6)	252 (49.4)	**0.001**		
Severe anaemia at delivery (< 7g /dL)^1^		21 (1.8)	23 (4.1)	**0.004**		
Peripheral *P falciparum* parasitemia at delivery^1^		23 (2.1)	10 (2.0)	0.861		
Placental malaria^2^		24 (2.4)	14 (3.0)	0.498		
Pregnancy outcomes						
	Live births	1105 (95.7)	500 (92.4)	**0.006**	0.96^a^ 0.96^b^	0.87–1.07 0.85–1.08
	Stillbirths^3^	30 (2.6)	27 (5.0)	**0.011**	1.97^a^ 3.34^b^	1.17–3.29 1.82–6.20
	Miscarriages^4^	20 (1.7)	12 (2.2)	0.492	1.26^a^ 1.67 ^b^	0.62–2.59 0.58–4.85
	Congenital malformations	11 (1.0)	5 (1.0)	0.961		
Maternal deaths		4 (0.3)	5 (0.9)	0.132		
Peripheral *P falciparum* parasitaemia one month after delivery in the mother		15 (2.0)	4 (1.0)	0.206		

* Proportions were compared using the chi-square test and continuous variables with the Student’s t test

^1^n (column percentage)

^2^Placental malaria was defined as parasites and/or pigment observed in a histologic examination and/or in an impression smear

^3^Stillbirth: foetal death that occurs after 20 complete weeks of gestation

^4^ Miscarriage: termination of pregnancy and expulsion of an embryo or of a foetus prior to 20 complete weeks of gestation (as estimated by measurement of fundal height).

^a^Unadjusted analysis

^b^Adjusted analysis by baseline variables (age, gestational age, literacy, gravidity, RPR, anaemia), study intervention, parasitaemia and reported ARV use at delivery.

Among HIV-infected women, a significant decrease in mean CD4+ T cells count (from 466.4 to 431.3 cells/μL, p = 0.013), and a significant increase in the proportion of women with undetectable HIV viral load (from 14.1 to 25.3%, p<0.001) was observed at delivery compared with baseline figures (Table 2). At the end of pregnancy, 21% of the women reported being on ART, 70% had received ARV drugs for PMTCT of HIV and 9% reported no ARV intake at all.

### Foetal and infant’s outcomes

Foetal anaemia was higher among infants born to HIV-infected (10.6%) than in those born to HIV-uninfected mothers (7.3%, p = 0.022). No differences were found in the proportion of cord blood parasitaemia, prematurity, mean birth weight, small for gestational age and other anthropometrical measures such as birth length and head circumference (Table 5). At one month of age (median visit age of 32 days), the proportion of infants with severely acute malnutrition was higher in those born to HIV-infected mothers (2.8%) than in infants born to HIV-uninfected women (1.3%) but the difference was not statistically significant (p = 0.064, Table 5). No significant differences were found in number of neonatal hospital admissions and neonatal deaths by HIV status of the mother (Tables 3 and 5). Among infants born to HIV-infected mothers, a total of 443 infants out of 500 live births (88.6%) had a HIV DNA PCR test done at four weeks of age (median 32 days). Forty one of them had a positive result, with a proportion of MTCT of HIV of 9.3%.

**Table 5:** Foetal and infants outcomes by HIV status of the mother.

Parameter	HIV-uninfected mother	HIV-infected mother	p- value*
***Birth***			
Cord blood parasitaemia	2/1051 (0.2)	1/483 (0.2)	0.945
Foetal haemoglobin (mean (SD)[n])	14.6 (2.0) [1049]	14.5 (2.4) [481]	0.213
Foetal anaemia (cord blood Hb<12.5 g/dL)	83/1143 (**7.3**)	57/540 (**10.6**)	**0.022**
Prematurity^1^	66/993 (6.7)	23/466 (4.9)	0.203
Mean birth weight (g, mean (SD)[n])	3030.1 (483.3) [1129]	3039.8 (526.6) [527)	0.721
Low birth weight (<2500 g)	107/1143 (9.4)	47/540 (8.7)	0.662
Small for gestational age (SGA)^2^	156/991 (15.7)	67/466 (14.4)	0.500
Length (cm, mean (SD)[n])	48.8 (13.8) [1089]	48.2 (2.9) [504]	0.331
Head circumference (cm, mean (SD)[n])	33.7 (1.8) [1089]	33.6 (2.1)	0.723
***Mortality***			
Early neonatal deaths^3^	17/1143 (1.5)	11/540 (2.0)	0.410
Neonatal deaths^4^	19/1143 (1.7)	15/540 (2.8)	0.129
***Month 1 visit***^***5***^			
Weight (g, mean (SD)[n])	4285.7 (772.3) [775]	4286 (825.7) [432]	0.993
Length (cm, mean (SD)[n])	52.7 (3.2) [772]	52.8 (3.2) [432]	0.203
Severe acute malnutrition^6^	10/779 (1.3)	12/434 (2.8)	0.064

* Proportions were compared using the chi-square test and continuous variables with the Student’s t test; Values are number n/N (column percentage) unless indicated otherwise

^1^Prematurity: birth before the beginning of the 37^th^ week (assessed by the Ballard score)

^2^ SGA was defined as an infant born ≥ 35 weeks' gestation and < 10^th^ percentile on the Fenton Growth Chart[37]

^3^Early neonatal deaths refers to a death of a live-born baby within the first seven days of life

^4^Neonatal deaths refers to a death of a live-born baby within the first 28 days of life

^5^Median age at the time of the visit was 32 days

^6^Severe acute malnutrition was defined as a weight for height (WAH) Z-score lower than -3 SD.

## Discussion

This prospective study of two cohorts of HIV-uninfected and HIV-infected pregnant women has shown that HIV infection continues to have a detrimental impact on maternal health and birth outcomes, contributing significantly to hospital admissions during pregnancy and to the burden of stillbirths. These effects of maternal HIV infection on maternal and neonatal health were observed despite the fact that ARV drugs have been roll-out in Mozambique for almost a decade. The findings confirm that anaemia,-including severe-, which constitutes an important cause of maternal and foetal morbidity, is more common in HIV-infected than in HIV-uninfected women. Similarly, maternal HIV infection had deleterious effects on the neonate’s health with an increased risk of foetal anaemia.

The majority of HIV-infected women were anaemic at ANC booking (71.5%) and nearly half of them were still anaemic at delivery (49.4%). These figures and those of the prevalence of severe anaemia are comparable to those reported in similar settings ranging from 50% to 83% [38–40]^,^[13, 41, 42]. Altogether, these findings indicate that HIV infection is a significant risk factor for maternal anaemia, which in turn affects negatively maternal and infant morbidity[13, 40, 43]. Moreover, anaemia in pregnancy has been associated with HIV infection progression and with adverse maternal and foetal outcomes [13, 39, 44]. The decrease in the proportion of women with anaemia from the first ANC visit to delivery in the two groups, may be explained by ferrous sulphate and folic acid supplementation provided free of charge at the monthly ANC visits in Mozambique[45]. Importantly, foetal anaemia was significantly higher in infants born to HIV-infected mothers. This finding supports the evidence indicating the association between maternal and foetal anaemia [46, 47]. Severe foetal anaemia has been associated with an increased risk of neonatal death[48]. These results indicate the urgent need to prevent and treat anaemia as early as possible in pregnancy with a special emphasis in HIV-infected women [45, 49].

The observed increased risk of stillbirths in HIV-infected women is consistent with previous studies [10, 11, 15]. In addition, although not statistically significant, we also found that HIV-infected women had more miscarriages than HIV-uninfected women [11, 50]. These results support the hypotheses suggesting that HIV infection constitutes either a direct cause or a marker of a complex interaction of related medical and social conditions that affect pregnancy[50, 51]. On the other hand, we found no effect of maternal HIV infection on birth weight, in contrast with previous studies from this same study area and from other southern African countries[9–11, 13, 52, 53]. Low birth weight is due to multifactorial factors that include socio-demographic and medical variables such as, constitutional factors, maternal age, gravidity, nutritional status, obstetric history, medical risks and antenatal care [54, 55]. Besides, no association was found between maternal HIV infection and prematurity, in agreement with previous studies conducted in Kenya and Tanzania [9, 53].

Due to their immunosuppressed status HIV-infected individuals are particularly vulnerable to infections[56]. Early studies showed that bacterial pneumonia, urinary tract infections and other infections are more common during pregnancy in HIV-infected women [51]. The findings of this prospective cohort study confirm previous observations indicating that HIV-infected pregnant women present an increased morbidity during pregnancy [2, 11, 32, 57]. Of notice is that the majority of the previous studies were conducted before the roll-out of antiretrovirals. In contrast, in the current study, over 90% of the HIV-infected women reported having received ARV drugs during this pregnancy (either as ART or PMTCT). However, despite a considerable proportion of women receiving ARV drugs, overall, the incidence of maternal morbidity was high among HIV-infected women. Overall, viral load decreased over the course of pregnancy which can be explained by the increase in the number of women on ARV drugs. The average level of CD4+ T cell counts also tended to decreased during pregnancy which can be related with the increased morbidity observed in the HIV-infected cohort. Overall, these findings call for the development of strategies that ensure adequate and high-quality ANC services to improve maternal health in this vulnerable group of pregnant women. For instance, the antenatal care package of HIV-infected women could include specific monitoring and preventive interventions to promptly detect possible causes of stillbirths and medical conditions that impair maternal morbidity.

Pregnant women have an increased susceptibility to malaria infection, which contributes to the morbidity of HIV-infected pregnant women living in endemic areas such as Malawi and Kenya [58, 59]. Nevertheless, in this study no differences were observed in the incidence of clinical malaria in pregnancy and in the prevalence of peripheral parasitemia at delivery between HIV-infected and uninfected women, in contrast with previous studies conducted in the same study area, Zimbabwe and Kenya [58, 60, 61]. This may be because all study women received effective malaria preventive tools (insecticide treated nets and IPTp) as part of the trial’s intervention, which may have resulted in a reduced exposure to the parasite reflected by the low prevalence of parasitaemia at delivery and placental infection [25, 26]. Notably, even if HIV-infected and uninfected women received different malaria prevention interventions.

Maternal HIV infection has been reported to be a risk factor for both maternal and infant mortality previously [2, 12, 62–64]. Although the small number of cases precluded reaching statistical significance, in this study there was also a trend towards increased proportion of both maternal and neonatal deaths among HIV-infected women.

The proportion of MTCT of HIV at four weeks of age was 9.3% and similar to that reported in another study conducted in this area a year earlier [52]. This proportion of MTCT reflects a slight decrease from the 12.4% figure reported in 2003–2006 [65]. Although small, this reduction in MTCT of HIV may be explained by the change in PMTCT guidelines in the last years in Mozambique from the administration of sd-NVP to the mother at the onset of labour and to the newborn within 72 hours of life in 2003, to AZT from 14 weeks of gestation in the current study; this led to the majority of HIV-infected women reporting at the time of delivery having received ARV drugs during this pregnancy. From 2013, guidelines to prevent MTCT of HIV in the country followed the latest WHO recommendation of providing life-long ARVs to pregnant women regardless of their immunological status (option B+) and are likely to reduce further MTCT rates [66].

Of notice, all study women were participating in a clinical trial and consequently they were under a close medical follow up which could constitute a study limitation. This could have underestimated the effect of maternal HIV infection on birth outcomes and infant’s health. However, over 1700 pregnant women contributed to this study and allowed us to perform robust analyses on the association between HIV infection and maternal and birth outcomes.

Another important limitation is that only self-reported information about ARV’s adherence was available in the study. Importantly, it has been shown that there is a low agreement between self-report and actual presence of antimalarial drugs in pregnant women from Uganda [67]. The use of self-reported information regarding adherence to treatment constitutes thus a limitation. Finally, the results presented in this work are part of a secondary analysis of data collected within the frame of two clinical trials which were designed to evaluate the efficacy of mefloquine for IPTp. Consequently, authors could not anticipate for collection of information about confounding factors (such as smoking and alcohol use among study participants) at the design level of the study for the analysis of maternal HIV infection impact on pregnancy and neonatal outcomes. This could also constitute a study limitation, however the aforementioned confounding factors are very rare in the study population and we do think they are affecting our results.

## Conclusions

We have documented that despite the introduction of antiretroviral drugs in the last decade in Mozambique, HIV infection continues to exact a major toll on maternal and infant’s health with a two-fold increased risk of maternal hospital admission and stillbirths in this area of the country. With the recently released WHO guidelines recommending ART initiation regardless of CD4+T cell counts to all HIV-infected individuals, it is expected that the number of people living with the infection will increase worldwide by improving their survival[68]. The amount of HIV-infected women of reproductive age in need of ART is also likely to increase, particularly in SSA, where the burden of disease concentrates and where resources are often scarce. Public health efforts should urgently be made to identify the barriers that are impeding improvements in the devastating effects of HIV in African women and their infants. Moreover, strategies aiming at increasing ART coverage and adherence during pregnancy and infancy should be prioritized in the global health agenda. Finally, monitoring the effects of HIV infection on both maternal and infant’s health should continue and be strengthen in order to evaluate and tailor the implemented preventive and therapeutic strategies.

## Supporting information

S1 Text Details of trials procedures. (DOCX)
