# Supplementary material for: Effects of HIV infection on maternal and neonatal health in southern Mozambique: A prospective cohort study after a decade of antiretroviral drugs roll out
Source: PLoS One. 2017 Jun 2;12(6):e0178134. doi: 10.1371/journal.pone.0178134 (PMC5456062; doi:10.1371/journal.pone.0178134)
Supplement: S1 Text — (DOCX) [file pone.0178134.s001.docx]

**S1. Text. Details of Trials procedures**

1. **Trial in HIV-uninfected pregnant women**

The study was designed as an open-label, randomized, three -arm trial to compare two-dose MQ with two-dose SP for IPTp, and to compare the tolerability of two different MQ administration regimens in the context of insecticide treated nets use. The three study arms were: 1) IPTp with SP, 2) IPTp with MQ (15 mg/kg) given once as a full dose, and 3) IPTp with MQ (15 mg/kg) split over two days.

Pregnant women of all gravidities attending an antenatal clinic (ANC) for the first time and who had not received IPTp during their current pregnancy were invited to participate in the study after provision of informed consent. **Inclusion criteria** were: permanent residence in the study area, gestational age ≤ 28 weeks, negative HIV-testing at recruitment, absence of history of allergy to sulfa drugs or mefloquine, absence of history of severe renal, hepatic, psychiatric or neurological disease, and of MQ or halofantrine treatment in the preceding 4 weeks. Gestational age was determined from fundal height measurement by bimanual palpation. Women not meeting inclusion criteria received standard antenatal care following national guidelines. Hemoglobin (Hb), HIV test and the syphilis rapid plasma reagin test (RPR) were assessed at the first antenatal visit as per local standard procedures. In Mozambique and Tanzania, HIV-infected women were invited to participate in a placebo-controlled trial evaluating MQ IPTp in women on daily cotrimoxazole prophylaxis[[1](#_ENREF_1)].

Women were encouraged to attend the ANC whenever they had any health complaint. Health care was free of charge and in general there was little availability of antimalarial drugs over the counter at all sites. A health facility-based passive surveillance system was established at each site to capture unscheduled visits of the study participants during the study follow-up. At each unscheduled visit, a standardized questionnaire was completed documenting signs and symptoms. Blood smears were prepared for malaria parasite examination and hemoglobin was measured if there were current or reported symptoms and/or signs suggestive of malaria. Clinical malaria episodes were treated with oral quinine or artemether-lumefantrine in the first and subsequent trimesters respectively for uncomplicated malaria, and with parenteral quinine for severe malaria. Solicited and unsolicited adverse events were assessed. The former was done by directed questioning of malaria related signs and symptoms during unscheduled visits, whereas the latter were assessed through open questioning during scheduled visits. Women who were withdrawn from the study received routine ANC treatment.

At delivery, women’s peripheral blood, cord blood and placental (biopsy and impression smears) samples were collected for hematological and parasitological evaluation. Newborns were weighed, and their gestational age at birth evaluated using the Ballard’s score. One month after the end of pregnancy, a capillary blood sample from the mother was collected for malaria parasite determination.

1. **Trial in HIV-infected pregnant women**

An individually randomized double-blind placebo-controlled trial was conducted to compare the efficacy of three monthly doses of MQ as IPTp with placebo-IPTp in HIV-infected pregnant women receiving daily CTXp in the context of LLITNs. The primary endpoint of the study was the prevalence of peripheral maternal malaria infection (microscopic or submicroscopic) at delivery.

Pregnant women of all gravidities attending the ANC for the first time and who had not received IPTp during their current pregnancy were given the opportunity to be included in the study after providing informed consent. Enrolled women were permanent residents in the area, had a gestational age ≤ 28 weeks, had a positive HIV-test at recruitment, absence of history of allergy to sulfa drugs or MQ, absence of history of severe renal, hepatic, psychiatric or neurological disease, and had not received MQ or halofantrine treatment in the preceding four weeks. In Mozambique and Tanzania, HIV-negative women were invited to participate in a randomized controlled trial evaluating the safety and efficacy of MQ IPTp compared to SP IPTp[[2](#_ENREF_2)]. Gestational age was determined from fundal height measured by bimanual palpation. Following national guidelines in place, HIV status was assessed after voluntary HIV counselling and testing with a HIV rapid test and the positive results confirmed with a second rapid test. Haemoglobin (Hb) and the syphilis rapid plasma reagin test (RPR) were assessed as part of routine ANC on fingerprick collected capillary blood, and 5mL venous blood was taken for CD4+T cell count and viral load determination. Women were recruited regardless of their immunosuppression level (as measured by CD4+T cell count) or whether or not they were already on anti-retroviral therapy (ART) for their own health.

Drug tolerability was assessed immediately and two days after intake by home visits of field workers. Women were encouraged to attend the study health facility whenever they had any health complaint. Health care was free of charge and in general there was little availability of antimalarial drugs over the counter at all sites. A health facility-based passive surveillance system was established at each site to capture unscheduled visits of study participants during study follow-up. At each unscheduled visit, a standardized questionnaire was completed documenting signs and symptoms. Blood smears were prepared for malaria parasite examination and haemoglobin was measured if there were current or reported symptoms and/or signs suggestive of malaria. Clinical malaria episodes were treated with oral quinine (first trimester) or artemether-lumefantrine (subsequent trimesters) for uncomplicated malaria; parenteral quinine was used for treatment of severe malaria. Solicited and unsolicited adverse events were assessed. The former was done by directed questioning of malaria related signs and symptoms during unscheduled visits, whereas the latter were assessed through open questioning during scheduled visits. HIV/AIDS management of study participants was provided by the local government health services according to national guidelines [[3-5](#_ENREF_3)]. Administration of antiretroviral drugs (ARVs) for Prevention of Mother to Child Transmission of HIV (PMTCT) or anti-retroviral treatment (ART) was registered in the study concomitant medication forms.

At delivery, a sample from the mother’s peripheral blood was collected for haemoglobin, CD4+T cell count, HIV viral load and malaria infection evaluation; cord blood and placental samples (biopsy and impression smears) were also taken, as well as blood onto filter paper for qPCR determinations. Newborns were weighed (including stillbirths) and measured and their gestational age at birth assessed using the Ballard’s score [[6](#_ENREF_6)]. Babies’ weights not captured at birth were estimated from weights obtained in the first week of life using a regression model [[7](#_ENREF_7)]. Six weeks after the end of pregnancy, a capillary blood sample from the mother was collected for malaria parasite determination. Infants born to study participants were followed until two months after birth to assess survival and general morbidity. Following national guidelines for PMTCT of HIV, a capillary blood sample was collected from the infant at six weeks of age onto filter paper for HIV PCR analysis.

References:

1. Gonzalez R, Desai M, Macete E, Ouma P, Kakolwa MA, et al. (2014) Intermittent Preventive Treatment of Malaria in Pregnancy with Mefloquine in HIV-Infected Women Receiving Cotrimoxazole Prophylaxis: A Multicenter Randomized Placebo-Controlled Trial. PLoS Med 11: e1001735.

2. Gonzalez R, Mombo-Ngoma G, Ouedraogo S, Kakolwa MA, Abdulla S, et al. (2014) Intermittent Preventive Treatment of Malaria in Pregnancy with Mefloquine in HIV-Negative Women: A Multicentre Randomized Controlled Trial. PLoS Med 11: e1001733.

3. Ministry of Health and Social Welfare-The United Republic of Tanzania (2007) National Guidelines Prevention of Mother-to-Child Transmission of HIV. Ministry of Health of The United Republic of Tanzania.

4. Ministry of Health- Republic of Kenya (2009) Guidelines for the prevention of Mother to Child transmission (PMTCT) of HIV/AIDS in Kenya. Ministry of Health - Kenya.

5. Ministério da Saúde-República de Moçambique (2013) Introduçao de Novas Normas para o seguimiento do paciente HIV positivo. Direcçao Nacional de Assistencia Médica Ministério da Saúde de Moçambique.

6. Ballard JL, Khoury JC, Wedig K, Wang L, Eilers-Walsman BL, et al. (1991) New Ballard Score, expanded to include extremely premature infants. J Pediatr 119: 417-423.

7. Greenwood AM, Armstrong JR, Byass P, Snow RW, Greenwood BM (1992) Malaria chemoprophylaxis, birth weight and child survival. Trans R Soc Trop Med Hyg 86: 483-485.
